# Increased platelet aggregation in hepatic tissue of metabolic-associated fatty liver disease—an observational study

**DOI:** 10.3389/fmed.2024.1503090

**Published:** 2025-01-06

**Authors:** Rongshan Fan, Yandong Li, Zeng Du

**Affiliations:** ^1^Department of Hepatology, Shenzhen Hospital of Integrated Traditional Chinese and Western Medicine, Shenzhen, Guangdong, China; ^2^Department of Pathology, Shenzhen Hospital of Integrated Traditional Chinese and Western Medicine, Shenzhen, Guangdong, China

**Keywords:** metabolic-associated fatty liver disease, hepatic tissue, platelet, aggregation, hepatic inflammation

## Abstract

**Objective:**

The study aimed to observe the quantity of platelet aggregation in the hepatic tissue of patients with metabolic-associated fatty liver disease (MAFLD) and its relationship with hepatic inflammation, fibrosis, and fatty degeneration.

**Methods:**

Clinical data of 55 patients with MAFLD and 25 patients without MAFLD, admitted to the Shenzhen Hospital of Integrated Traditional Chinese and Western Medicine from December 2020 to May 2022, were retrospectively analyzed. Liver tissue obtained by liver biopsy underwent routine pathological examination. Immunohistochemical staining with CD63 antibody was performed to label platelets in the liver tissue. Clinical, liver pathology, and immunohistochemical staining data of the study subjects were statistically analyzed using unpaired t-test.

**Results:**

The quantity of platelet aggregation in the hepatic tissue was higher in MAFLD patients than in non-MAFLD patients and was related to the degree of hepatic inflammation but not to the degree of hepatic fibrosis or fatty degeneration.

**Conclusion:**

The quantity of platelet aggregation in hepatic tissue was increased in patients with MAFLD and was related to the degree of hepatic inflammation,

## 1 Introduction

Nonalcoholic fatty liver disease (NAFLD) is a type of metabolic stress–induced liver injury closely associated with insulin resistance and genetic susceptibility. It represents the hepatic manifestation of metabolic syndrome, encompassing a spectrum of diseases including simple steatosis (NAFL), nonalcoholic steatohepatitis (NASH), and associated liver fibrosis, cirrhosis, and hepatocellular carcinoma. The Asia-Pacific Association for the Study of Liver officially renamed it as metabolic-associated fatty liver disease (MAFLD) in June 2020 (1). Furthermore, MAFLD is closely linked to metabolic syndrome, type 2 diabetes, atherosclerotic cardiovascular diseases, and colorectal tumors (2).

Platelets, derived from megakaryocytes in the bone marrow, are involved in hemostasis, thrombosis, obesity, atherosclerosis, metabolism, and stroke. Studies on an MAFLD model showed that platelets participate in the recruitment of immune cells and cytokine induction in the liver, leading to liver injury. Lipid toxicity is speculated to cause platelet aggregation, adhesion, and activation in the liver, triggering immune inflammatory responses and activating stellate cells, thereby promoting the development of liver fibrosis and hepatocellular carcinoma. Antiplatelet drugs such as aspirin and clopidogrel can interrupt this cascade reaction, thus preventing and treating NASH (3–6).

Although animal experiments demonstrated the involvement of platelets in MAFLD pathogenesis, direct evidence is needed to determine whether liver platelet aggregation, leading to hepatic steatosis, indeed occurs in patients with MAFLD. Therefore, we investigated whether the quantity of platelet aggregation in the liver of MAFLD patients increases, aiming to provide direct evidence for the involvement of platelets in MAFLD pathogenesis.

## 2 Materials and methods

### 2.1 Study subjects

Employing a retrospective study method, 84 patients admitted to the Shenzhen Hospital of Integrated Traditional Chinese and Western Medicine from December 2020 to May 2022 were selected. Of these, 55 patients who met the diagnostic criteria for MAFLD, which was confirmed by liver biopsy, were categorized into the test group, whereas 29 patients diagnosed with non-MAFLD based on liver biopsy were assigned to the control group. The diagnostic criteria provided in the “Guidelines for the Prevention and Treatment of Nonalcoholic Fatty Liver Disease (2018 Update)” were used (2).

Inclusion criteria comprised (1) the age of 30–45 years; (2) no history of alcohol consumption or alcohol intake of <210 g per week for males and < 140 g per week for females in the past 12 months; (3) liver imaging findings consistent with the radiological diagnostic criteria for diffuse fatty liver; (4) liver histopathological changes consistent with the pathological diagnostic criteria for fatty liver disease.

Exclusion criteria were alcoholic liver disease, genotype 3 hepatitis C, autoimmune hepatitis, hepatic glycogenosis, and conditions leading to fatty liver, including taking medication (tamoxifen, amiodarone, sodium valproate, methotrexate, glucocorticoids, etc.), total parenteral nutrition, inflammatory bowel disease, celiac disease, hypothyroidism, Cushing’s syndrome, beta-lipoprotein deficiency syndrome, lipodystrophy-associated diabetes, and Mauriac syndrome.

### 2.2 Data collection

Subjects’ names, genders, ages, alanine aminotransferase (ALT) levels, complete blood counts, and liver pathology examinations were recorded. Additionally, intrahepatic platelet aggregation was detected and quantified.

Pathological Examination Method: Before the procedure, routine blood tests and coagulation function tests were conducted for all patients to exclude contraindications such as coagulation disorders. Under ultrasound guidance, liver tissue was obtained via percutaneous liver biopsy using a 16G disposable needle (Bard Company, United States). Specimens were immediately fixed in neutral formalin for examination, followed by embedding in paraffin, consecutive sectioning, and staining with hematoxylin and eosin, Sirius red, Masson’s trichrome, and reticulin staining. Two experienced pathologists from the Pathology Department of the Shenzhen Hospital of Integrated Traditional Chinese and Western Medicine reviewed the pathological sections.

Liver Pathology Scoring Criteria: Liver pathology scoring was performed referring to the “Guidelines for the Prevention and Treatment of Nonalcoholic Fatty Liver Disease (2018 Update)” (2). Liver tissue was scored for fatty degeneration, with inflammatory lesions graded from 0 to 4 (G0–4) based on portal, periportal, and lobular inflammation. Fibrotic lesions were graded from 0 to 4 (S0–4) based on the extent of and their impact on hepatic microcirculation. Fatty degeneration was graded from 0 to 4 (F0–4) based on the degree of steatosis, ballooning degeneration, lobular inflammation, and fibrosis (F0, no steatosis; F1, simple steatosis; F2, lobular inflammation and ballooning degeneration, fibrosis stage 1; F3, lobular inflammation and ballooning degeneration, fibrosis stages 2–3; F4, lobular inflammation and ballooning degeneration, fibrosis stage 4).

Detection of Intrahepatic Platelet Aggregation Quantity: Immunohistochemical staining was performed using the immunoenzymatic method with CD63 recombinant rabbit monoclonal antibody (Thermofisher). Experimental procedures strictly followed the instructions provided with the reagents.

Platelet Counting Method: Cells showing brownish cytoplasm and platelet morphology were considered positive cells. Five random fields were observed under low magnification, with images captured under high magnification. The area of each field was 1 mm^2^, and the number of positive cells in each field was counted to calculate an average.

### 2.3 Statistical methods

Statistical analysis was performed using GraphPad 5.0 software. Normally distributed quantitative data were expressed as x ± s (mean ± standard deviation). The chi-square test was used for inter-group comparison of count data, while the unpaired t-test was used for inter-group comparison of measurement data. One-way analysis of variance was used for comparison among multiple groups. The Mann–Whitney U test was used for inter-group comparison of non-normally distributed semi-quantitative data. A *p* < 0.05 indicated statistical significance.

## 3 Results

### 3.1 General information

The MAFLD group comprised 55 patients, including 50 males and 5 females with an average age of 36.98 years. The non-MAFLD group consisted of 29 patients, including 27 males and 2 females with an average age of 38.48 years. Gender and age showed no statistically significant differences between the two groups (Table 1).

**Table 1:** Comparison of demographic data.

Item	MAFLD group (*n* = 55)	Non-MAFLD group (*n* = 29)	Test method	Statistic	*p* value
Gender (%)			Adjusted χ^2^ test (two-sided)	X^2^ = 0.146	*p* > 0.05
Male	51	27			
Female	4	2			
Age (years)	37.10 ± 0.61	39.10 ± 0.75	*t*-test	T = 1.978	*p* = 0.0511

### 3.2 Comparison of ALT levels, peripheral blood platelet parameters, and liver tissue lesions

ALT levels did not significantly differ between the groups. The comparison of various parameters of peripheral blood platelets between the groups, including platelet count (PLT), mean platelet volume (MPV), plateletcrit (PCT), platelet distribution width (PDW), and platelet-large cell ratio (P-LCR), showed no statistically significant differences. Comparison of liver tissue inflammation grades and fibrosis stages between the two groups also showed no statistically significant differences (Table 2).

**Table 2:** Comparison of ALT levels, peripheral blood parameters, and liver tissue lesions in study groups.

Item		MAFLD group (*n* = 55)	Non-MAFLD group (*n* = 29)	Test method	Statistic	*p* value
ALT (U/L)		56.55 ± 9.98	48.13 ± 13.94	*t*-test	*T* = 0.492	*p* = 0.624
PLT (×10^9^/L)		260.70 ± 26.26	188.80 + 11.64	*t*-test	*T* = 1.480	*p* = 0.143
MPV (fL)		10.38 ± 0.155	10.70 ± 0.244		*T* = 1.154	*p* = 0.252
PCT (%)		0.21 ± 0.01	0.196 + 0.01	*t-*test	*T* = 1.662	*p* = 0.100
PDW (fL)		16.18 ± 0.42	16.22 ± 0.72	*t*-test	*T* = 0.615	*p* = 0.540
P-LCR		28.38 ± 1.05	30.63 ± 1.69	*t*-test	*T* = 1.191	*p* = 0.237
Inflammatory lesions	G0	0	1	Mann Whitney U test	*U* = 755.000	*p* = 0.075
	G1	8	11			
	G2	39	17			
	G3	2	0			
	G4	0	0			
Fibrotic lesions	S0	2	0	Mann Whitney U test	*U* = 736.500	*p* = 0.086
	S1	19	17			
	S2	14	7			
	S3	19	5			
	S4	1	0			

### 3.3 Liver tissue platelet count results

**Figure 1:**
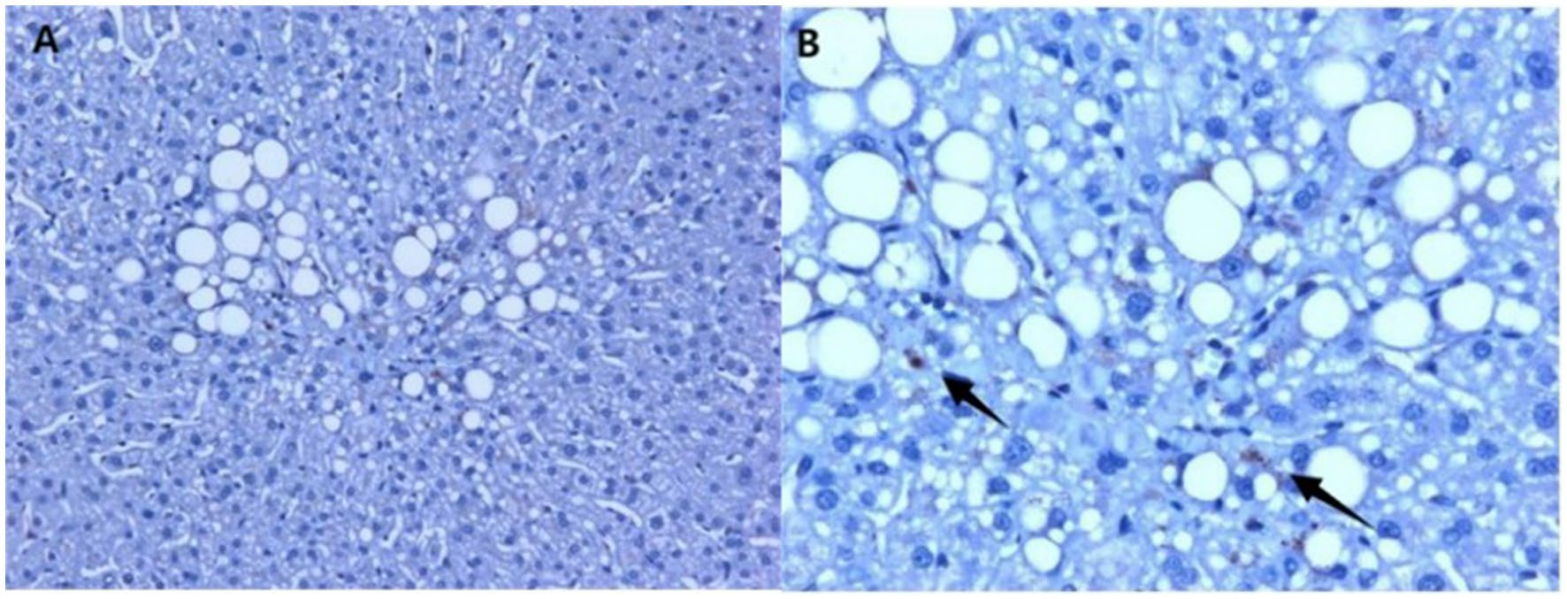
Immunohistochemical staining using CD63 in the liver tissue of MAFLD patients, showing hepatic steatosis and platelets in the liver tissue. **(A)** 20× magnification; **(B)** 40× magnification (arrows indicate platelets, which are visualized in the brownish color).

**Figure 2:**
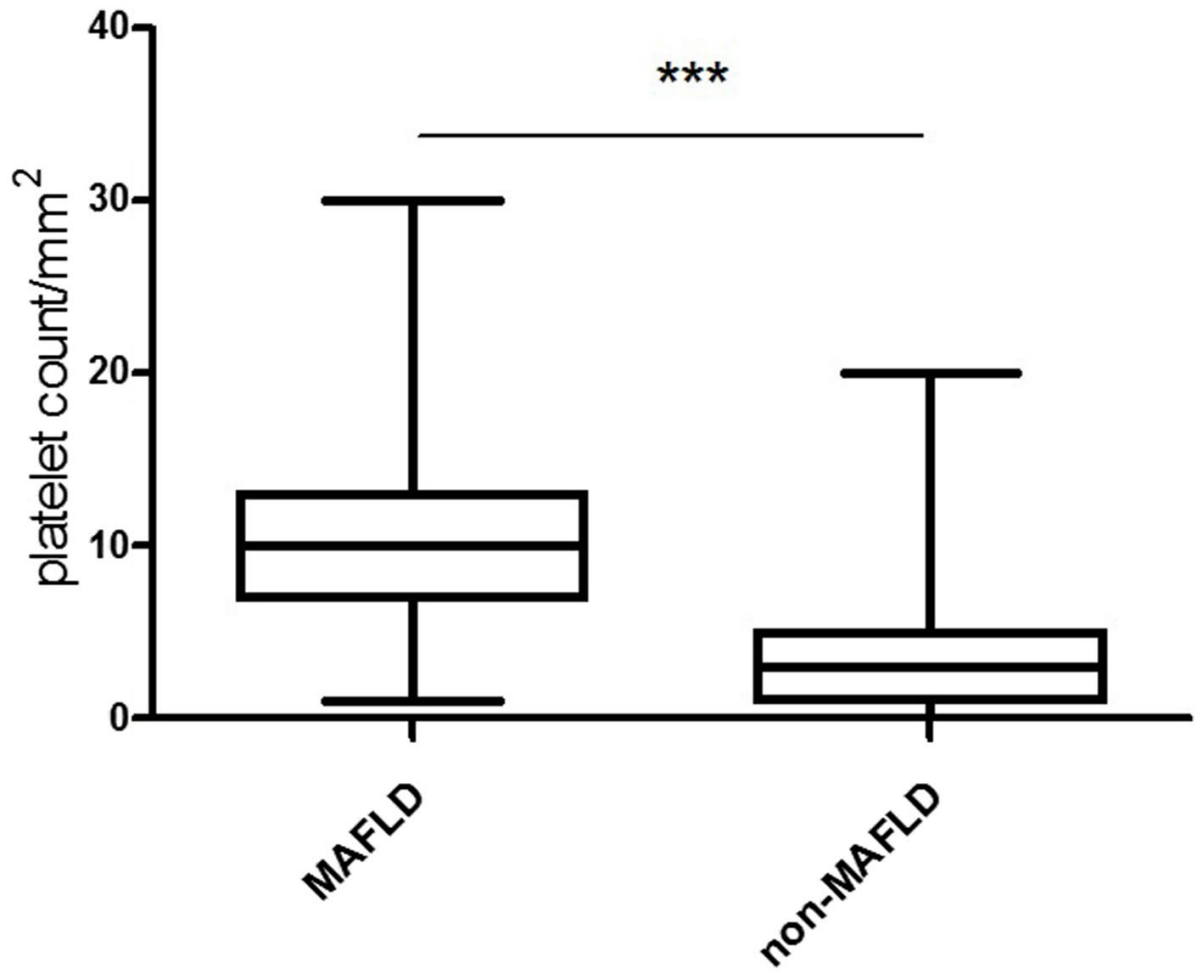
Platelet count in the liver tissue of study subjects (Figure 1). The platelet aggregation quantity was 10.60 ± 0.82/mm^2^ in the MAFLD group and 4.45 ± 1.02/mm^2^ in the non-MAFLD group, with T = 4.548, indicating a statistically significant difference (*p* < 0.0001).

In the liver tissue obtained through liver biopsy, platelets were clearly visible through CD63 immunohistochemical staining. The platelet aggregation quantity in the liver tissue was 10.60 ± 0.82/mm^2^ in the MAFLD group and 4.45 ± 1.02/mm^2^ in the non-MAFLD group. Unpaired *t*-test analysis showed T = 4.548, *p* < 0.0001, indicating a statistically significant difference (Figures 1, 2).

### 3.4 Correlation between liver tissue platelet aggregation quantity and hepatic inflammation, fibrosis, and fatty degeneration

In the MAFLD group, liver tissue inflammation was classified into G1 grade (*n* = 11) and G > 1 grade (*n* = 44). Comparing the platelet aggregation quantity between the two groups revealed a higher platelet aggregation quantity in the G > 1 grade group, with a statistically significant difference. According to liver tissue fibrosis, the MAFLD group was classified into S1 (*n* = 19), S2 (*n* = 13), and S3–S4 stage (*n* = 23). No statistically significant differences were observed regarding platelet aggregation quantity among the three groups. According to fatty degeneration, the MAFLD group was classified into F1 (*n* = 44) and F2–3 grade (n = 11). No statistically significant differences were observed regarding platelet aggregation quantity among the two groups (Table 3).

**Table 3:** Comparison of liver tissue platelet counts between different inflammation, fibrosis, and fatty degeneration grades in the MAFLD group.

Item	Grades	Number	Platelet counts in liver tissue /mm^2^(x ± s)	Test method	Statistic	*p value*
Inflammation grades	G1	*n* = 11	6.727 ± 0.8954	*t*-test	*T* = 2.467	*p* = 0.0173
	G > 1	*n* = 44	11.72 ± 1.038			
Fibrosis grades	S1	*n* = 19	9.895 ± 1.420	One-way analysis of variance	*F* = 0.1241	*p* = 0.8836
	S2	*n* = 13	10.380 ± 1.814			
	S3–S4	*n* = 23	10.940 ± 1.541			
Fatty grades	F1	*n* = 44	9.639 ± 0.9043	*t-*test	*T* = 0.6516	*p* = 0.5180
	F2–F3	*n* = 11	10.820 ± 1.387			

## 4 Discussion

No significant differences in age, gender, and ALT levels were identified between the two groups, excluding the influence of these factors on study results.

Additionally, our study found no significant differences in peripheral blood PLT, MPV, PCT, PDW, and P-LCR between MAFLD and non-MAFLD patients. This is consistent with the findings of Malehmir et al. (6), who demonstrated that liver tissue platelet aggregation increased in an MAFLD mouse model, with peripheral blood platelet count, activation, and aggregation abilities remaining unchanged.

However, Michalak et al. showed that MAFLD patients had lower MPV and PCT in peripheral blood compared to healthy controls, with considerable differences observed in liver fibrosis stages between the two groups (7). In contrast, our study found no significant differences in liver pathological fibrosis staging between MAFLD and non-MAFLD patients. The population studied in the previous literature comprised of patients with MAFLD identified based on ultrasound findings. These subjects did not undergo liver biopsy; hence, the degree of liver tissue lesions in the two groups was unknown. In our study, individuals with MAFLD were selected based on liver biopsy results, showing no significant differences in the degree of inflammation and fibrosis between the groups. Therefore, the different selection of study objects might be the reason for different platelet parameters in the peripheral blood.

Our study showed that the quantity of platelets in the liver tissue of MAFLD patients increased, which is consistent with the results of studies on MAFLD animal models (8) and MAFLD patients (9). This finding provides direct evidence for the involvement of platelets in MAFLD pathogenesis. Thus, platelets are non-resident cells that enter the liver in the early stages of NASH, forming the pathological basis of NASH. Similarly, increased platelet aggregation in the liver has been observed in chronic hepatitis, cholangitis, and drug-induced liver damage, in which activated platelets adhere to endothelial cells and Kupffer cells, inducing an inflammatory response (10).

Our study demonstrated that platelets are associated with the degree of hepatic inflammation, with increasing platelet aggregation quantity with worsening hepatic inflammation. Contrary to expectations, our study found no correlation between platelet quantity and the degree of fibrosis, although platelets are involved in the development of liver fibrosis. This may be explained by the fact that platelet infiltration into liver tissue is an initiating factor in the early stages of fibrosis. Additionally, the mechanism of developing fibrotic lesions is complex and long, involving multiple factors. Furthermore, other studies indicated that platelets can secrete hepatic growth factor to inhibit the activation of hematopoietic stem cells, reducing collagen synthesis and TGF-β1 expression, thereby suppressing hepatic fibrosis (11). Therefore, in the progression of MAFLD to liver fibrosis and even liver cancer, the dual anti- and pro-fibrotic effects of platelets need further exploration. Additionally, although platelets are involved in hepatic steatosis, our study showed no correlation between platelet quantity and the degree of fatty degeneration in liver tissue. This finding might also be explained by the fact that platelet infiltration into liver tissue is an initiating factor in the early stages of the pathology, and the quantity is nonlinearly correlated with the degree of fatty degeneration (5).

In conclusion, our study found that the quantity of platelet aggregation in the liver tissue of MAFLD patients was increased, indicating that the early entry of platelets into the liver forms the basis for hepatic steatosis. Therefore, platelet regulation may have potential significance for MAFLD treatment. Antiplatelet therapy can improve imaging, pathological, and biochemical indicators of MAFLD patients (12, 13). Accordingly, the first suggestion of a clinically beneficial role of aspirin was published in 2014 (14). In a prospective study of patients with biopsy-proven MAFLD, daily aspirin use was associated with less severe histologic features of MAFLD and NASH and a lower risk for progression to advanced fibrosis with time (13). Hence, antiplatelet drugs could prevent MAFLD progression. Further research is needed to determine the mechanism of action of drugs and potential specific targets. Accordingly, clinical trials on antiplatelet interventions in MAFLD patients may be conducted in the future. Of course, limited by the number of cases, the study results need to be further confirmed, and the relationship between the platelet quantity in liver tissue and the degree of liver fibrosis needs to be further studied in more cases.

## Data Availability

The original contributions presented in the study are included in the article/supplementary material, further inquiries can be directed to the corresponding author.
